# Recombinant nucleases CEL I from celery and SP I from spinach for mutation detection

**DOI:** 10.1186/1472-6750-7-29

**Published:** 2007-06-01

**Authors:** Maxim Pimkin, Elena Caretti, Adrian Canutescu, Jeffrey B Yeung, Heather Cohn, Yibai Chen, Catherine Oleykowski, Alfonso Bellacosa, Anthony T Yeung

**Affiliations:** 1 Basic Science, Fox Chase Cancer Center, Philadelphia, PA, USA; 2 Population Science, Fox Chase Cancer Center, Philadelphia, PA, USA

## Abstract

**Background:**

The detection of unknown mutations is important in research and medicine. For this purpose, a mismatch-specific endonuclease CEL I from celery has been established as a useful tool in high throughput projects. Previously, CEL I-like activities were described only in a variety of plants and could not be expressed in an active form in bacteria.

**Results:**

We describe expression of active recombinant plant mismatch endonucleases and modification of their activities. We also report the cloning of a CEL I ortholog from *Spinacia oleracea *(spinach) which we termed SP I nuclease. Active CEL I and SP I nucleases were expressed as C-terminal hexahistidine fusions and affinity purified from the cell culture media. Both recombinant enzymes were active in mutation detection in *BRCA1 *gene of patient-derived DNA. Native SP nuclease purified from spinach is unable to incise at single-nucleotide substitutions and loops containing a guanine nucleotide, but the recombinant SP I nuclease can cut at these sites.

**Conclusion:**

The insect cell-expressed CEL I orthologs may not be identical to their native counterparts purified from plant tissues. The present expression system should facilitate further development of CEL I-based mutation detection technologies.

## Background

Nucleases of the S1 family are widely used as tools for probing single-stranded regions of DNA and RNA [1-3] as well as for the removal of single-stranded regions from dsDNA [3,4]. One class of plant homologs of S1, represented by CEL I from celery, are particularly capable of efficient cutting at single base substitutions and loops [5-7]. Several CEL I-based mutation detection techniques have been developed [8-12]. They are relatively simple yet highly reliable and capable of detecting a mutation in pools of several DNA samples. Adaptation of this approach to the Tilling method of recovering chemically derived mutations at target regions [10,13] has allowed CEL I to contribute to many plant genetics programs [14-17], as well as zebrafish [18,19], drosophila [20], and mouse ES cells research [21]. Moreover, it is beginning to be successfully applied to programs of disease mutation detection [6,22-27]. A CEL I ortholog, CEL II nuclease, is the principal component of the SURVEYOR Mutation Detection Kits (Transgenomic, Inc.) [7].

The P1 nuclease of *Penicillium citrinum *is a close ortholog of the S1 nuclease. Although its crystal structure has provided important clues to the mechanism of phosphodiester bond cleavage and single-stranded oligonucleotide binding [28], the applicability of this model for CEL I orthologs has not been tested. Several important questions remain. How is the wide range of mismatch substrates recognized by CEL I? What determines the pH optima for RNase and DNase activities [7,9,29]? How can these enzymes be engineered into even better mutation-detection tools? Clearly, a better mechanistic understanding of single-strand specific nucleases is needed to answer these questions. Development of an expression system for this class of enzymes will be an important step in this direction.

Most single-strand specific nucleases are extracellular glycoproteins containing one or more disulfide bridges per monomer to confer high enzyme stability [3]. Such proteins are hard to express in an active form in a heterologous system which often fails to provide the correct pattern of posttranslational modifications. Indeed, our attempts to express active CEL I in various prokaryotic hosts have not succeeded.

In the present study we employed a baculovirus system for expression and site-directed mutagenesis studies of enzymes of the CEL I family. Active CEL I nuclease was purified from the cell culture media and used for detection of single-base substitutions in patient-derived DNA. We also report the cloning, expression and site-directed mutagenesis of the cDNA of a close homolog of CEL I nuclease from spinach, which we termed SP I. We show that the properties of recombinant CEL I and SP I nucleases may be different from their native counterparts purified from plant tissues.

## Results

### Cloning of the SP I nuclease cDNA

A nuclease isolated from spinach, called SP, is a particularly intriguing CEL I ortholog, showing properties intermediate between CEL I and S1. It has a strong preference for AT-rich regions, yet is able to cut single-base mismatches and has a neutral pH optimum. Unlike CEL I, it is unable to recognize mismatches with guanine bases at the mismatched site [30]. We cloned this CEL I ortholog from spinach mRNA. We called this putative nuclease SP I to distinguish the properties of this clonally purified form from the native SP nuclease preparations that may contain more than one homolog that are very difficult to separate during native enzyme purification. The SP I mRNA sequence was deposited to GenBank under accession no. [GenBank:EF032908].

**Figure 1:**
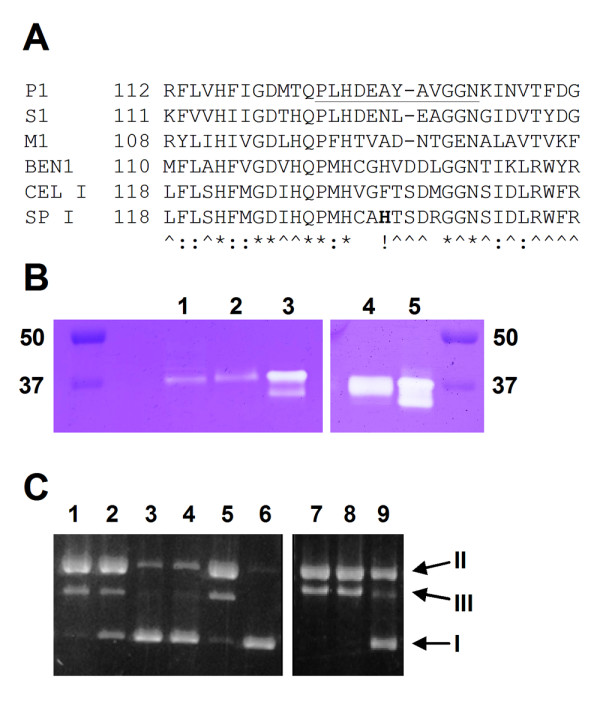
**Cloning, expression and purification of recombinant nucleases**. (A) A ClustalW alignment of the SP I amino acid sequence with homologous sequences. Amino acid numbering is given with respect to the primary structure of mature P1. P1 nuclease of *Penicillium citrinum *[GenBank:P24289]; S1 nuclease of *Aspergillus oryzae *[GenBank:AAB20216]; M1 nuclease of *Mesorhizobium loti *[GenBank:BAB52626]; BEN1 nuclease of *Hordeum vulgare *[GenBank:BAA28942]; CEL I nuclease of *Apium graveolens *[GenBank:AAF42954]; SP I nuclease of *Spinacia oleracea *[GenBank:ABK34453]. The nucleotide binding sequence of P1 is underlined [28]. Symbols: *, identity; :, strong similarity; ., weak similarity; ^, resudies identical in CEL I and SP I. (B) Detection of single-strand DNase activities after in-gel enzyme refolding. Lane 1 and 2, Ni^2+ ^affinity-purified SP I^wt ^and SP I^H135A ^nucleases, respectively; lanes 3 and 5, native CEL nuclease purified from celery, after the MonoQ step; this sample is a combination of CEL I and CEL II nucleases [5]; lane 4, recombinant CEL I nuclease purified on a Ni^2+ ^affinity column. (C) Induction of single-strand specific activity in infected Sf9 cells detected by RF-I nicking assay. Lanes 1–4, 0.1 μl cell extract was used in 20 μl reaction; lanes 7–9, 1 μl cell culture media in 20 μl reaction; lanes 1 and 7, CEL I nuclease expression; lanes 2 and 8, SP I^wt ^nuclease expression; lanes 3 and 9, cells infected with an "empty" control vector containing no nuclease gene; lane 4, extract of non-infected cells; lane 5, native CEL nuclease purified from celery; lane 6, uncut pUC19 DNA. I, RF-I supercoiled plasmid DNA; II, RF-II nicked circular plasmid DNA; III, RF-III linearized plasmid DNA.

SP I and CEL I amino acid sequences are 71.1% identical. Amino acid sequence alignment of SP I with other known S1-like nucleases reveals that all residues identified by structural studies [28] as crucial for binding of the three Zn^2+ ^atoms and for catalysis are preserved in SP I. Interestingly, the nucleotide binding site shows less sequence conservation (Figure 1A). A significant divergence was observed in a fragment within a loop located close to the ligand, comprising residues 134–139 in SP I and 127–132 in P1, respectively. The H135 residue is one of the most prominent sequence features within this fragment of the SP I nuclease, compared with CEL I and P1. To test if H135 is important for the SP I mismatch-specific nuclease activity, we produced a recombinant virus expressing a H135A mutant of the SP I nuclease.

### Recombinant expression of CEL I and SP I

Infection of a Sf9 cell culture with recombinant viruses containing the CEL I or SP I genes under control of a constitutive promoter resulted in accumulation of a single-strand specific nuclease activity both in the culture media and cell extract (Figure 1C). This activity adhered to a Ni^2+ ^affinity column and was eluted with 150 mM imidazole. A single major nuclease band was observed when the partially purified nuclease preparations were separated on a SDS PAGE, in-gel refolded, and stained for single-strand specific nuclease activity (Figure 1B). This activity co-migrated with a native CEL I control (purified from celery), implying that the recombinant enzyme contains a similar quantity of N-linked glycans. We also detected the recombinant protein by a Western blot experiment with an anti-hexahistidine monoclonal antibody (not shown). The Ni^2+ ^affinity column-purified nucleases were stable on ice for at least a week and infinitely stable when stored in 50% glycerol at -20°C. The enzyme activities were reduced by freezing/thawing cycles, decreasing by roughly 50% after each cycle (data not shown).

### Test of recombinant nucleases in mutation detection

**Figure 2:**
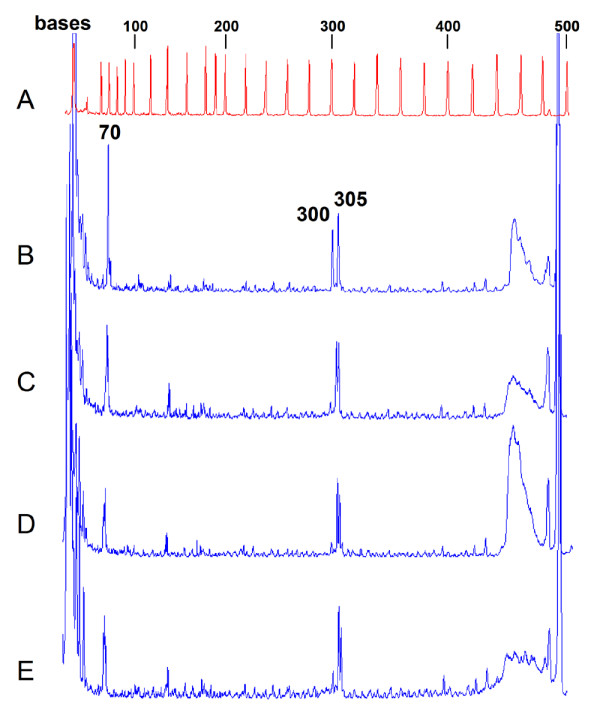
**Recombinant CEL I, SP I^wt ^and SP I^H135A ^nucleases' action on a multi-mismatch PCR substrate**. Simultaneous detection of three SNPs in one 490 bp PCR product of *BRCA1 *gene derived from a heterozygous patient. The DNA strand labeled with Cy5.5 fluorescent dye is shown. Unprocessed chromatograms are shown in full-scale display. (A) Molecular weight standards, Beckman Coulter. (B) Native CEL nuclease purified from celery, pooled fractions after MonoQ step [5]. (C) Recombinant CEL I. (D) SP I^wt^. (E) SP I^H135A^. The 70, 300 and 305 nt long CEL I reaction products correspond to *BRCA1 *nucleotide substitutions 2430 C → T, 2201 T → C and 2196 G → A, respectively.

**Figure 3:**
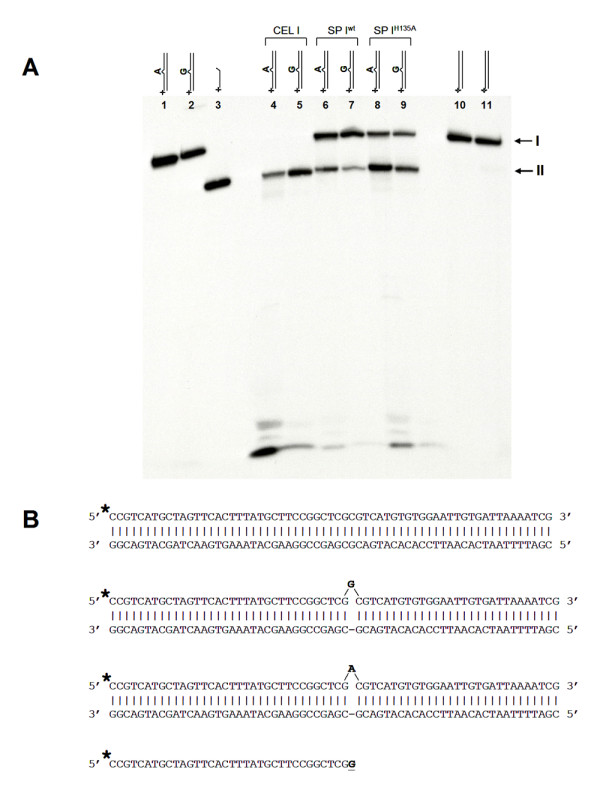
**Incisions at single nucleotide extrahelical loops by SP I^wt ^and SP I^H135A^**. (A) Autoradiogram of a denaturing PAGE. Lanes 1–2, intact substrates with no endonuclease treatment; lane 3, control oligonucleotide corresponding to the product of an incision 3' of the mismatched base; lanes 4–5, A and G extrahelical loop substrates incubated with CEL nuclease purified from celery; lanes 6–9, A and G extrahelical loop substrates incubated with recombinant SP I^wt ^and SP I^H135A ^nucleases; lanes 10–11, perfect duplex substrate incubated with SP I^wt ^and SP I^H135A ^nucleases, respectively. I, full-length oligonucleotide substrate labeled at the top strand. II, products of an incision at the mismatched nucleotide. The lower molecular weight bands at the bottom of the gel are mononucleotides and short oligonucleotides resulting from the 5' to 3' exonuclease activity of the native and recombinant enzymes [7]. (B) Design of a perfect duplex substrate, mismatched heteroduplex substrates and a control oligonucleotide corresponding to the CEL I reaction product. The location of the ^32^P label is shown with an asterisk.

**Figure 4:**
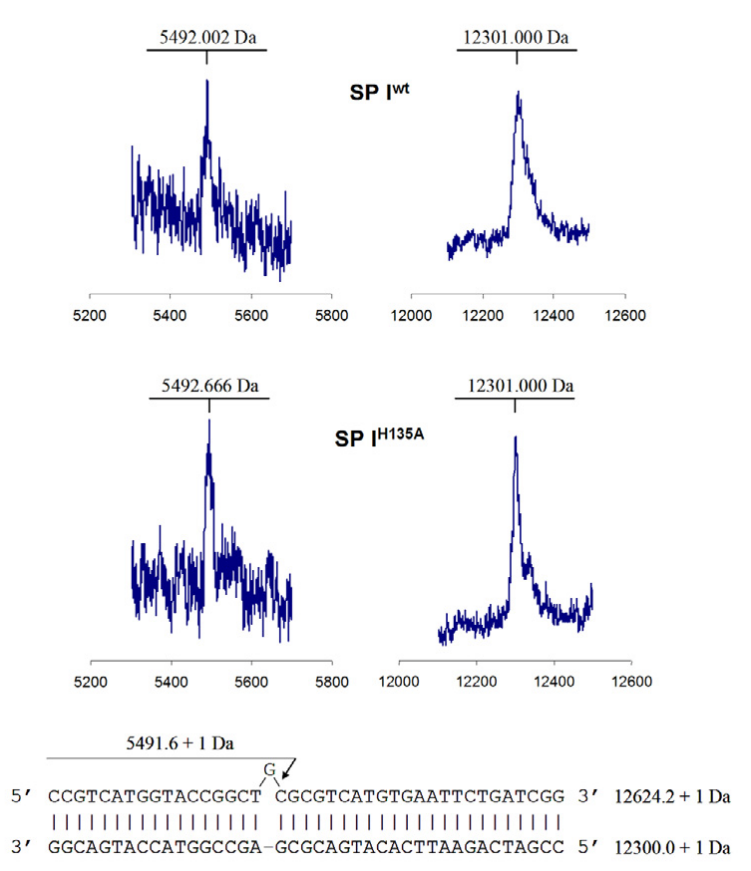
**Detection of specific incisions 3' of the mismatched G nucleotide by mass spectrometry**. The full length substrate (bottom strand) and the incision product peaks are shown. Design of the heteroduplex substrate used and the expected mass of the product are presented at the bottom. "+1 Da" refers to a single-protonated oligonucleotide ion.

To test the efficacy of our recombinant nuclease preparations in mutation detection, a highly polymorphic section of exon 11 of *BRCA1 *was used as the substrate [8]. A PCR product derived from one patient contained three single base pair polymorphisms as revealed by a control experiment with CEL I purified from celery. This substrate is challenging because of multiple single-base substitutions in close proximity to each other, a quality that would render many mutation detection techniques ineffective [9]. While the mismatches 2196 G → A and 2430 C → T were well detected by all our recombinant nuclease preparations (Figure 2), little cutting of the nucleotide substitution T → C at position 2201 of *BRCA1 *was observed, reflected by the low signal from the 300 nt long fragment (Figure 2). We also observed an additional 303–304 nt peak which may have originated from processing of the 305 nt peak by CEL I and SP I exonuclease activities. Control experiments have indicated efficient cutting of T → C at position 2201 of *BRCA1 *by native CEL I. Since this result was reproduced in all our recombinant SP I and CEL I preparations, it indicates that the mismatch sequence preference and possibly the balance between the exo- and endonuclease activities of the expressed enzymes is slightly different from their native counterparts purified from plant tissues. Surprisingly, unlike its native counterpart [30], the recombinant SP I was capable of introducing nicks specifically 3' of an extrahelical G nucleotide (Figure 3). This result was reproduced on several preparations of SP I and confirmed by mass spectrometry analysis of the incised heteroduplex substrates to exclude the possibility of a non-specific action of SP I on its substrate (Figure 4), further indicating that mismatch preferences of CEL I orthologs can be modified by recombinant expression.

SP I ^H135A ^was an active nuclease, with mismatch recognition properties similar to those of the wild type SP I (Figures 2 and 3), indicating that H135 is dispensable for the mismatch nuclease activity of SP I.

## Discussion

Expression of a protein in a heterogeneous host followed by purification is an important step in the study of protein function, allowing the unequivocal assignment of a function to a polypeptide. Plants may contain multiple CEL I-like nucleases which often co-purify due to aggregation by lectins in plant extracts [5]. Celery has at least two active CEL I orthologs, termed CEL I and CEL II [5], with similar mismatch cutting activities but with striking differences in pH optima and Mg^2+ ^requirements [7]. A BLAST search of the *Arabidopsis *genome yields several CEL I-like ORFs [9]. Recombinant expression of individual homologs in a heterologous system as shown in this report overcomes the cross-contamination and will facilitate their characterization. In fact, enhanced enzyme purity is a likely explanation for the modification of the properties of recombinant CEL I and SP I enzymes. The apparently altered sequence specificity of recombinant CEL I may be the result of removing the CEL II enzyme normally present in CEL I preparations from celery as demonstrated in Figure 1B. In line with this interpretation, the mismatch recognition preferences of mixtures of CEL I and CEL II nucleases have been reported to differ slightly from those of homogenous preparations of CEL I nuclease or CEL II nuclease [7].

Single-strand specific nucleases have been reported to vary widely in their efficiency with different substrates depending on the sequence context [30], size of the single-stranded region within a double-stranded substrate [31] and pH [29]. Based on the latter two criteria, two groups of S1 homologs may be distinguished: S1 orthologs and CEL I orthologs [9]. S1 orthologs are represented by fungal nucleases S1 from *Aspergillus oryzae *and P1 from *Penicillium citrinum*, plant mung bean nuclease, and recently, recombinant prokaryotic M1 nuclease [29]. These enzymes are highly specific for single-stranded nucleic acids, have acidic pH optima, and are essentially inactive at alkali pH. They cut double-stranded DNA at relatively large distorted regions, such as the site of an insertion mismatch of three nucleotides or larger or at AT rich regions of double-stranded DNA, but not at base-substitution mismatches. In contrast, CEL I orthologs, represented by CEL I from celery, are active DNases at both acidic and alkali pH ranges, may be stimulated by or require Mg^2+^, and cut all DNA mismatches including single base substitutions at the phosphodiester bonds specifically 3' of the mismatch nucleotides. No structural explanations for this divergence of nuclease properties have been published and, to this end, it is unclear to what extent the structure of P1 may be relevant for making conclusions about CEL I orthologs [9]. The present expression system may help address this question by expression and characterization of other single-strand specific nucleases of the S1 and CEL I family and with the use of a site-directed mutagenesis approach.

One possibility for the surprising ability of our recombinant SP I nuclease to cut at a mismatch containing a G residue is that the cloned cDNA product may not represent the dominant CEL I-like activity found in spinach extracts. This possibility can be distinguished by obtaining sufficient quantities of native SP protein for amino acid sequence determination. Alternatively, SP I expressed in insect cells may assume a more relaxed structure, allowing binding of guanine nucleotides. Such relaxation could result from different number and positioning of disulfide bridges, glycosylation patterns and/or folding environments. Neither native nor recombinant CEL I has been characterized with respect to their disulfide bonding patterns.

## Conclusion

In the present study, we reported recombinant expression of plant mismatch endonuclease CEL I and a newly cloned CEL I ortholog, SP I, in a baculovirus system. Active enzymes were expressed as C-terminal hexahistidine fusions, purified from cell culture media using metal affinity chromatography and used for detection of mutations in *BRCA1 *gene of patient-derived DNA. Unlike its native counterpart purified from plant tissues, recombinant SP I nuclease was able to nick the phosphodiester bond 3' of an extrahelical guanine residue. Thus, recombinant expression of CEL I orthologs may result in modification of their activities due to enhanced enzyme purity and/or different pattern of post-translational modiciations.

Recent years have seen a major increase in the use of CEL I for genetic variance detection. CEL I has become an indispensable instrument in applications where high throughput and capability to recognize all mutations are critical. All of the mentioned applications would benefit from further improvement of CEL I mismatch-cutting properties. For instance, it would be highly desirable to eliminate the 5' to 3' directional endonuclease activity which results in the removal of a 5'-label and prohibits extended incubation of a substrate with the nuclease. The present CEL I expression system may open a new avenue for developing engineered enzymes with enhanced mutation detection properties.

## Methods

Native CEL nuclease was purified from celery stalks according to the published procedure [5] and, like most purified CEL nuclease preparations, is a mixture of CEL I and CEL II enzymes (Figure 1B). RF-I nicking experiments and in-gel enzyme refolding followed by activity staining were performed as described [29].

### Cloning of the cDNA of SP I nuclease mRNA

Total RNA was prepared from store-bought fresh spinach (*Spinacia oleracea *Melody hybrid) leaves using the phenol SDS procedure for plant RNA extraction as described [32]. Stratagene's Pro-Star First Strand RT-PCR kit was used to synthesize first-strand cDNA. We used the CEL I nuclease amino acid sequence [GenBank:AAF42954] [5] to construct two pairs of degenerate primers that allowed amplification of SP I cDNA in two segments. The resulting products were cloned in a TA vector using the TA Cloning^® ^Kit (Invitrogen), and sequenced with the use of vector-specific primers. By using 5' and 3' RACE technology (Stratagene), sequences of the 3' and 5' SP I mRNA coding regions were obtained. A pair of primers (sequences 5' TTTCAATGTCGCGTTCTACT and 5' AGTCCTAAACATTGGAAGCC) and Pfu DNA polymerase were used to amplify the entire protein-coding region of SP I cDNA which was cloned in the pCR^®^2.1 TA vector (Invitrogen), yielding the pSP plasmid. The entire insert in the pSP plasmid was sequenced using vector-specific primers and the SP I cDNA sequence was deposited to GenBank under accession No. [GenBank:EF032908].

### Construction of the expression plasmids pAcSP, pAcSPmut and pAcCELI

A pair of primers (5' GGGCTCGAGATGACGCGATTATATTCTGTGTTCTTTCT and 5' GGAGGTACCGAATTCAGTGGTGGTGGTGGTGGTGTTCTTCTGCCAAAGAATGATCTGCGGA) was used to amplify the CEL I gene that had been cloned from celery mRNA in our previous study [5]. The SP I nuclease gene was amplified from the pSP plasmid using the following pair of primers: 5' GGGCTCGAGATGTCGCGTTCTACTTGTTTTGTTTC and 5' GGAGGTACCGAATTCAGTGGTGGTGGTGGTGGTGTTCTTCTGTGGCGACTACCATTGCTT. The restriction nuclease recognition sites and hexahistidine-coding sequences are underlined. The PCR products were digested with *Kpn*I and *Xho*I restriction enzymes and cloned in pAcSG2 baculovirus transfer vector (PharMingen). The resulting plasmids were termed pAcSP with a length of 6420 bp and pAcCELI plasmid with a length of 6411 bp. The recombinant nucleases produced were C-terminal hexahistidine fusions with calculated molecular masses 35,339 Da for the SP I nuclease and 34,976 Da for the CEL I nuclease, respectively.

The QuikChange mutagenesis reaction (Stratagene) to create the pAcSPmut vector expressing a H135A mutant of SP I nuclease was conducted in accordance with manufacturer's recommendations. (For convenience, amino acid numbering throughout the manuscript is given with respect to the putative mature proteins starting with N-terminal tryptophan and lacking signal peptides. H135 of putative mature SP I corresponds to H158 of the expressed sequence.) A pair of complementary oligonucleotides was used: 5' GATATTCATCAGCCAATGCATTGCGCGGCGACCAGCGATAGAGGAGGAAATTC and 5' GAATTTCCTCCTCTATCGCTGGTCGCCGCGCAATGCATTGGCTGATGAATATC. The Ala135 codon substituting the His codon of wild-type SP I is underlined.

### Protein expression and purification

All tissue culture procedures, co-transfection and virus amplification were done according to Pharmingen recommendations [33]. Briefly, monolayer Sf9 cultures were co-transformed with an expression plasmid and BaculoGold Bright linearized DNA. The recombinant virus produced was amplified twice. Fluorescent microscopy was used to inspect the cells for the presence of GFP which was the marker of infection. Flow cytometry was used to assess virus titers by an end-point dilution assay. Monolayer cultures of Sf9 cells grown in TNM-FH medium were used for protein expression. In a typical experiment 5 × 10^7 ^cells were infected with 6 ml of ~1 × 10^8 ^pfu/ml amplified virus stock. Three days after infection the cell extract and culture medium were analyzed for plasmid nicking activity. Hexahistidine-tagged proteins were then purified on a HIS-Select Ni^++ ^column (Sigma) from the cell culture media. The crude medium was passed through a 0.22 μm filter (Millipore), diluted two-fold with Equilibration/Wash buffer (50 mM Tris-HCl, pH 7.6, 300 mM NaCl, and 10 μM ZnCl_2_), and loaded on a column that had been equilibrated with the same buffer. After loading, the column was washed with Equilibration/Wash buffer and then with 50 mM Tris-HCl, pH 7.6, 300 mM NaCl, 10 μM ZnCl_2_, 5 mM imidazole. Nucleases were eluted with 50 mM Tris-HCl, pH 7.6, 300 mM NaCl, 10 μM ZnCl_2_, and 150 mM imidazole.

### BRCA1 mutation analysis on a capillary DNA sequencer

Human genomic DNA, purified from blood samples from patients participating in the Margaret Dyson/Family Risk Assessment Program, was obtained from the Fox Chase Cancer Center Biorepository with approval of the Institutional Review Committee (protocol #00-824). A pair of primers specific for exon 11.4 of the *BRCA1 *gene (sequences 5' CCTTCCCTAGAGTGCTAAC and 5' CCCACCTAATTGTACTGAA) were synthesized with Cy5 fluorescent label at the 5' end of the forward primer and Cy5.5 label at the 5' end of the reverse primer. Twenty μl PCR reactions included 2 μl 10× PCR buffer (Applied Biosystems), 5% DMSO, 2 mM MgCl_2_, 0.2 mM each dNTP, 0.0375 μM each fluorescent primer, 100 ng human genomic DNA template and 0.2 U AmpliTaq Gold DNA polymerase (Applied Biosystems). The thermal cycling protocol consisted of a 5 min initial denaturation step at 94°C, followed by 35 cycles of (denaturation at 94°C for 10 s, annealing at 55°C for 20 s and elongation at 72°C for 1 min). PCR amplification products were heated to 94°C and gradually cooled to 4°C to allow formation of heteroduplexes. The resulting fluorescent substrates were incubated with recombinant nuclease preparations at 45°C for 60 min in CEL I reaction buffer (20 mM HEPES, pH 7.5, 3 mM MgCl_2_, 10 mM KCl), purified using the CEQ8000 ethanol-glycogen cleanup procedure (Beckman) and separated on Beckman CEQ8000 Genetic Analysis System according to the manufacturer's protocol.

### Incisions by SP I^wt ^and SP I^H135A ^at single nucleotide extrahelical loops

The oligonucleotides for making the mismatched substrates were synthesized in the Fox Chase Cancer Center Fannie E. Rippel Biotechnology Facility and PAGE-purified. DNA heteroduplex substrates containing A or G extrahelical loops were constructed by annealing a 5'-labeled oligonucleotide to a partially complementary cold nucleotide as shown in Figure 3B. Prior to annealing, the singe-stranded oligonucleotides were labeled at the 5'-termini with T4 polynucleotide kinase and [γ-^32^P]ATP. One hundred fmol of a heteroduplex substrate was incubated with recombinant nuclease preparations in 20 μl reaction volume in CEL I reaction buffer (20 mM HEPES, pH 7.5, 10 mM KCl, 3 mM MgCl_2_). Taq DNA polymerase (0.5 Units) was added to stimulate the mismatch-specific activity of CEL I and SP I [6]. The reactions were performed at 45°C for 1 h, terminated with formamide and analyzed on a denaturing PAGE gel. Autoradiography was used to visualize radioactive bands.

### Detection of the site of incision by mass spectrometry

Unlabeled heteroduplex oligonucleotide substrate was constructed as shown in Figure 4. The CEL I mismatch endonuclease assay was performed as described above. The reaction was stopped with EDTA and the reaction products were desalted using C18 ZipTip (Millipore Corporation) before mass spectral analysis. The ZipTip pre-concentration and AnchorChip (Bruker Daltonics) technique for MALDI spotting were employed. 3-HPA (3-hydroxypicolinic acid) was used as the MALDI matrix. 0.5 μl of 1% 3-HPA, 0.1% diammonium hydrogen citrate was applied onto 400 μm spot on the anchor plate and allowed to dry. 0.5 μl of desalted and pre-concentrated oligonucleotide reaction products was applied onto the matrix crystals. Mass spectra were acquired on a MALDI-TOF-MS Reflex IV instrument (Bruker Daltonics) in a linear delayed pulse ion extraction mode. The oligonucleotides were desorbed and ionized by a nitrogen pulsed laser with a wavelength of 337 nm. Internal calibration was carried out using singly and doubly charged ions from the full-length oligonucleotide substrate.

## Abbreviations

PAGE, polyacrylamide gel electrophoresis; PCR, polymerase chain reaction; ORF, open reading frame; SDS, sodium dodecyl sulfate; GFP, green fluorescent protein; pfu, plague-forming units; MALDI, matrix-assisted laser desorption/ionization; MS, mass spectrometry; TOF, time-of-flight.

## Authors' contributions

MP carried out protein expression and purification, CEQ8000 assays, participated in molecular cloning and participated in drafting of the manuscript. JBY, HC and CO participated in the molecular cloning and tissue culture procedures. EC and AB carried out the experiments with ^32^P-labeled substrates. YC performed mass analyses of oligonucleotides. AC performed the sequence alignment. ATY designed the study, provided general coordination and participated in drafting of the manuscript. All authors read and approved the final manuscript.
